# Supplementary figures and images for: Low-level overexpression of wild type TDP-43 causes late-onset, progressive neurodegeneration and paralysis in mice
Source: PLoS One. 2022 Feb 3;17(2):e0255710. doi: 10.1371/journal.pone.0255710 (PMC8812852; doi:10.1371/journal.pone.0255710)

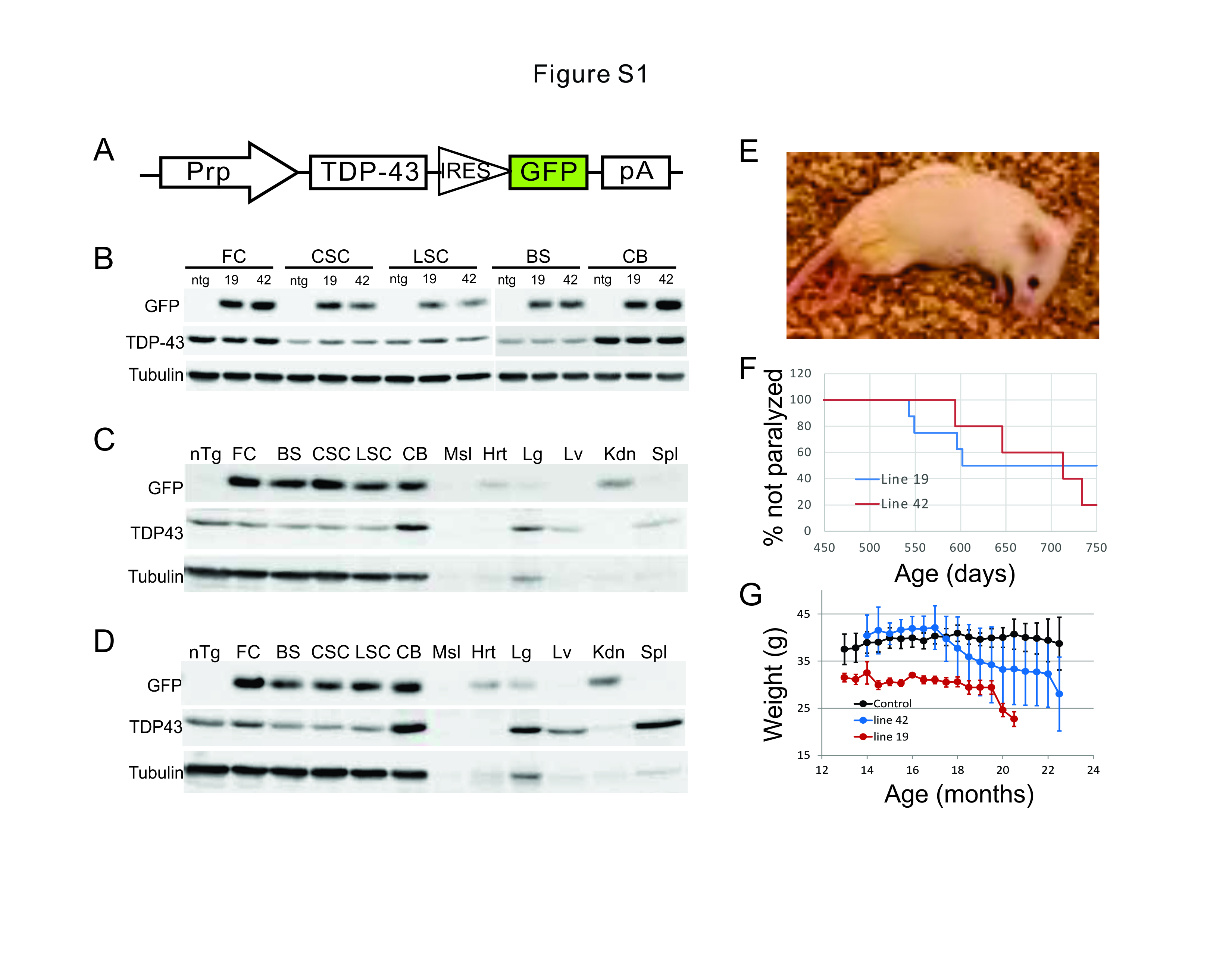

Supplement: S1 Fig — (A) A cDNA encoding mouse TDP-43 and EGFP linked by internal ribosome entry site (IRES) were inserted into the backbone of MoPrp.Xho plasmid to generate the Prp-mouse TDP-43 transgene. The construct was composed of the following elements in linear succession: the Prp promoter, mouse wild-type TDP-43, IRES, EGFP gene, and poly A signal. This construct (Prp-TDP-43) expresses TDP-43 and GFP separately. (B) Western blot showed that the transgene was expressed in all the CNS regions in transgenic lines 19 and 42. FC, frontal cortex; CSC, cervical spinal cord; LSC, lumbar spinal cord; BS, brainstem; CB, cerebellum. (C) A survey of the transgene expression in different organs in transgenic line 19 showed that the transgenes were predominately expressed in CNS. Low levels of expression were also detected in heart (Hrt), lung (Lg) and kidney (Kdn). Other tissues are muscle (Msl), liver (Lv), and spleen (Spl). (D) A survey of the transgene expression in different organs in transgenic line 42. Similar to line 19, the transgenes were predominately expressed in the CNS. The samples in B, C, and D were prepared from animals between 55 and 65 days old. (E) A mouse at the paralysis stage. Its limbs were paralyzed, and the mouse lost its local motion capability. (F) Monitoring small cohorts of mice from lines 19 and 42 showed late-onset paralysis but incomplete penetrance from both lines up to 750 days. (G) Progressive weight loss in the aged mice of the two transgenic lines. The animal numbers are 3 to 4 for line 42, 3 to 5 for line 19, and 3 to 15 for non-transgenic (nTg) control mice at different age points. For the TDP-43 mice, only animals that develop paralysis were included in the weight plots. Error bars are standard errors. (JPG) [file pone.0255710.s001.jpg]

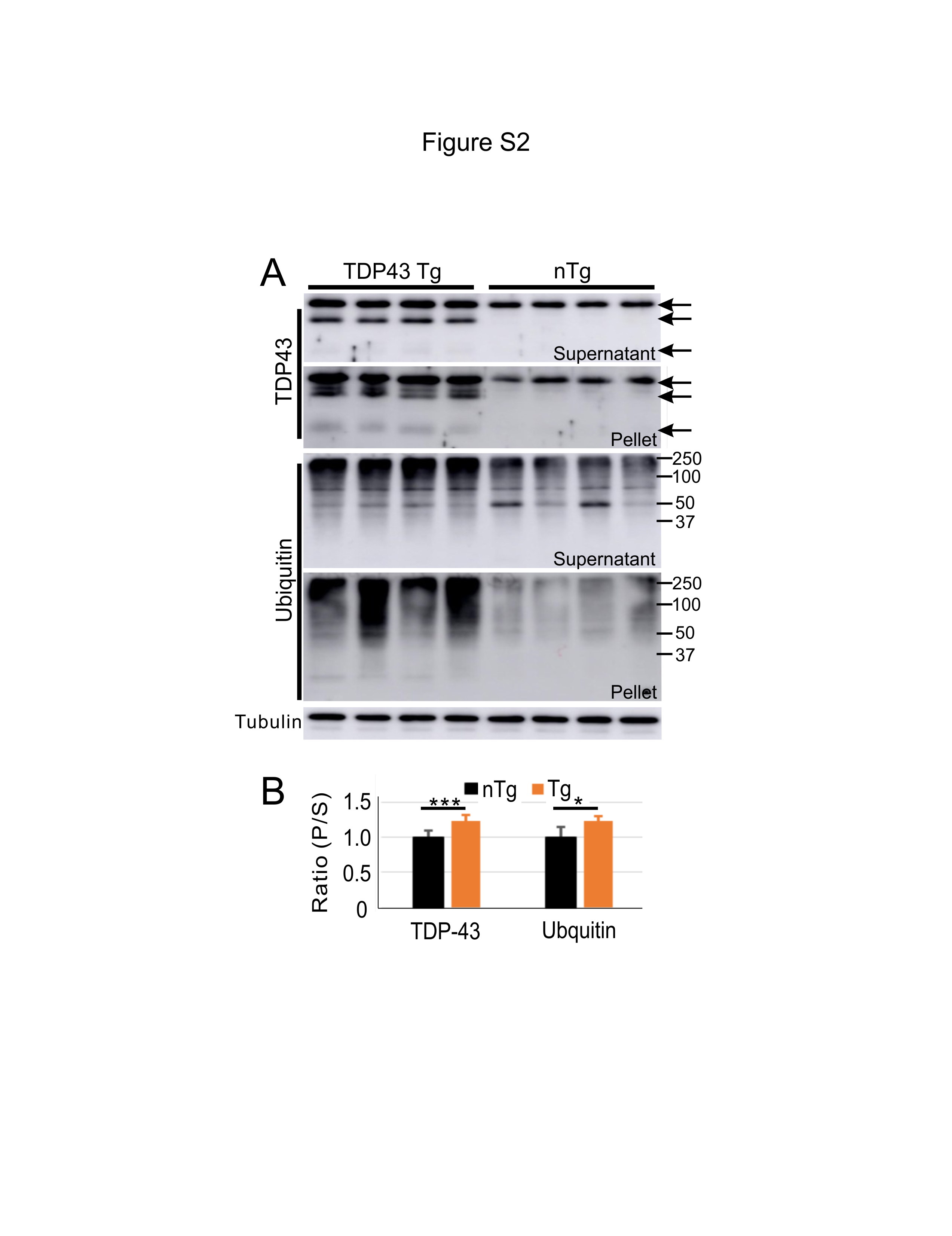

Supplement: S2 Fig — (A) Western blot of detergent-soluble and insoluble TDP-43 and ubiquitinated proteins extracted from lumbar spinal cords of paralyzed Tg mice and age-matched nTg controls. Each lane was loaded with proteins from one animal. Arrows point to TDP-43 and its 35 KD and 25 KD fragments. Numbers on the right indicate molecular weights in kD. (B) Relative ratios of band intensity of the pellet over the supernatant. Bars represent averages of 7 nTg and 8 Tg animals in the TDP-43 quantification and of 4 animals in both nTg and Tg groups in the ubiquitin quantification. Student t test was used to compare between Tg and nTg mice. * indicates p<0.05 and *** p<0.001. (JPG) [file pone.0255710.s002.jpg]

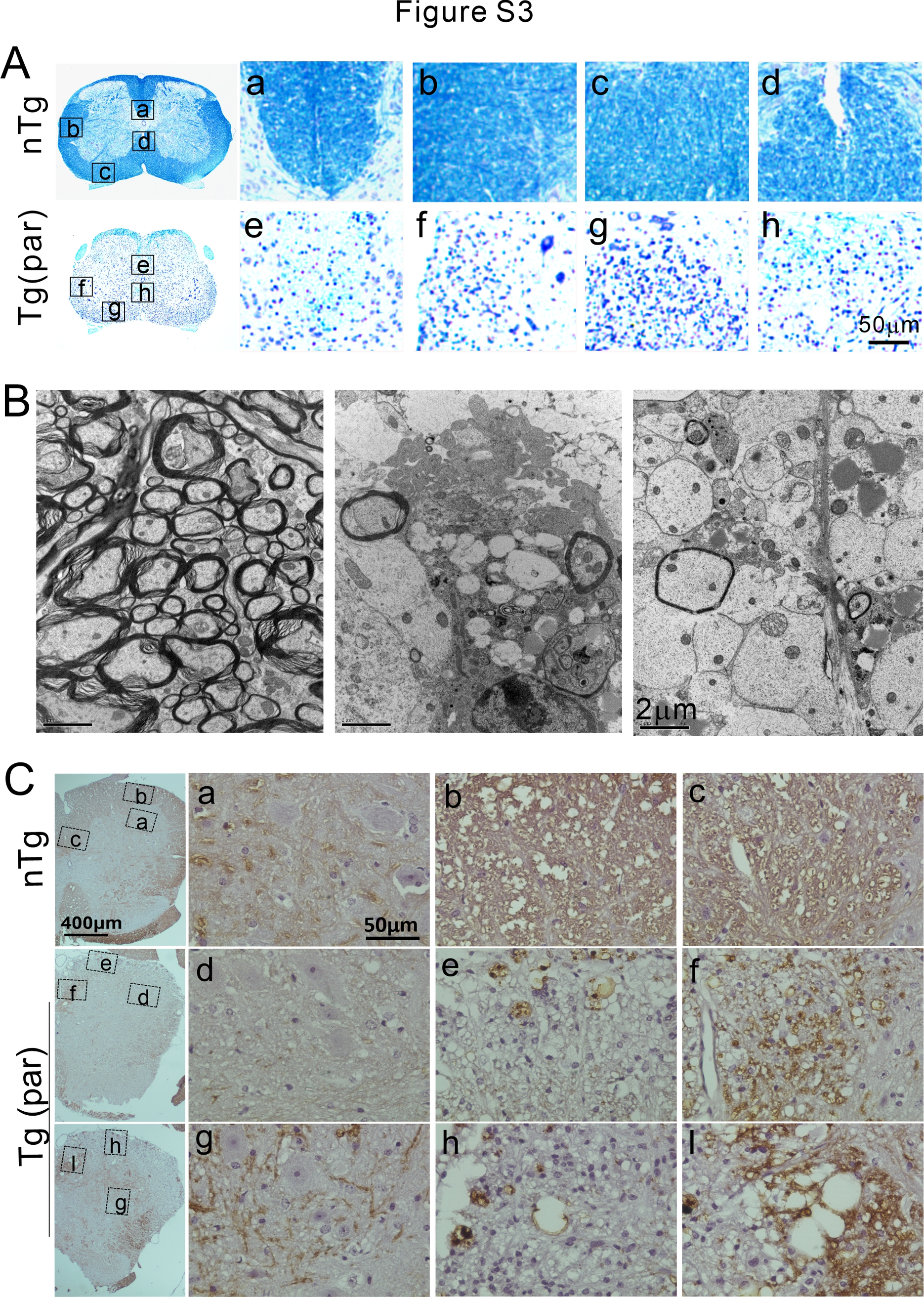

Supplement: S3 Fig — (Aa, e) cortical spinal track, (Ab, f) lateral funiculus, (Ac, g) ventral funiculus, (Ad, h) anterior commissure. (B) Electron microscopic images of ventral funiculus from a nTg mouse (left) and a Tg mouse (middle and right). Notice very few axons were wrapped by myelin in the Tg mice. (C) Immunohistochemistry staining for MBP in spinal cord ventral horn (a, d, g), ventral funiculus (b, e, h), and anterior commissure (c, f, i). (TIF) [file pone.0255710.s003.tif]

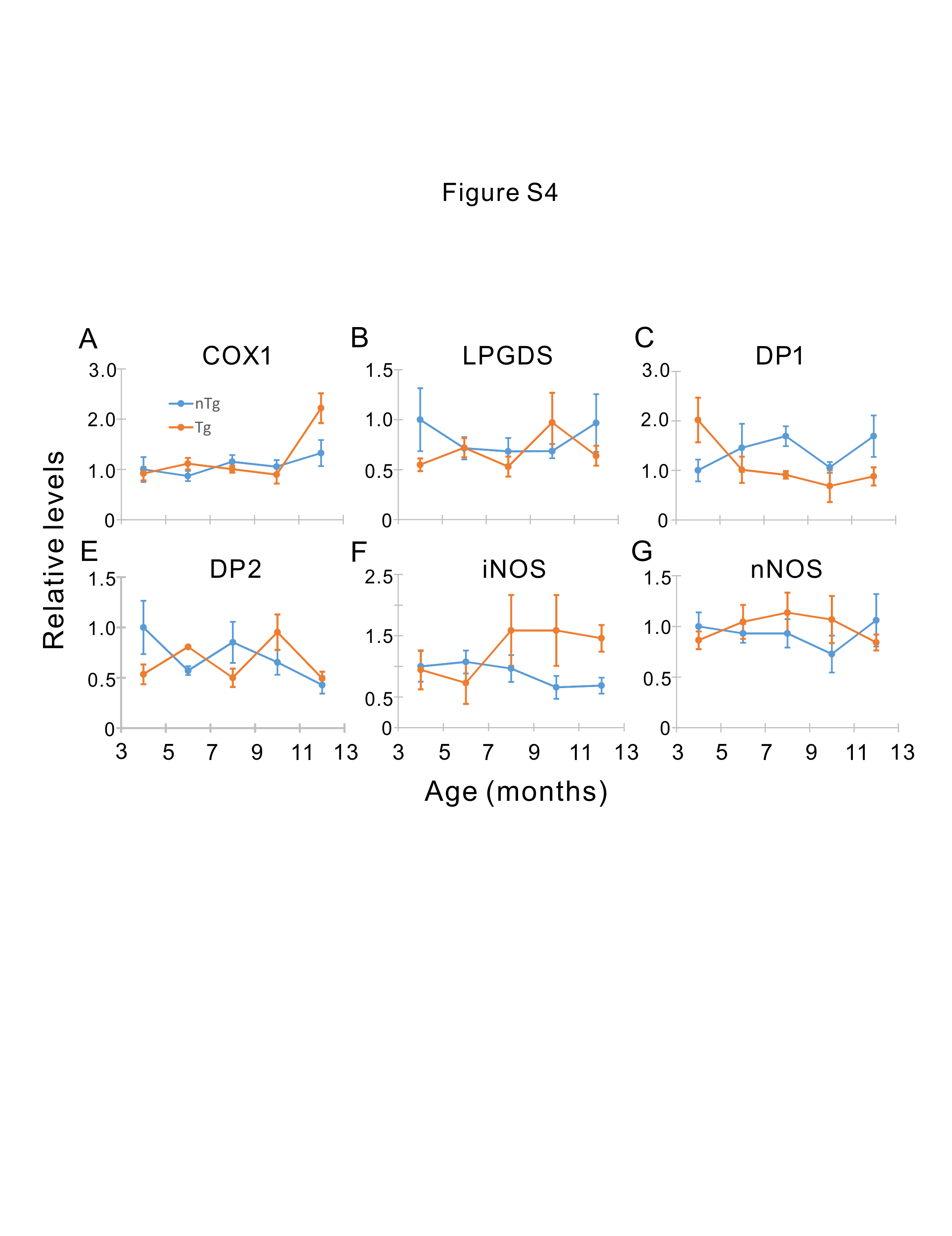

Supplement: S4 Fig — (A) cyclooxygenase 1 (COX-1 or PTGS1). (B) lipocalin type of prostaglandin synthase (LPGDS). (C) prostaglandin D2 receptor (DP1). (D) prostaglandin D2 receptor 2 (DP2). (E) inducible nitric oxide synthase (iNOS). (G) neuronal nitric oxide synthase (nNOS). Student t tests with Bonferroni correction were used to compare between the Tg and nTg mice at different ages. n = 3–8 at each age for both groups. No significance was found (p > 0.05). (JPG) [file pone.0255710.s004.jpg]

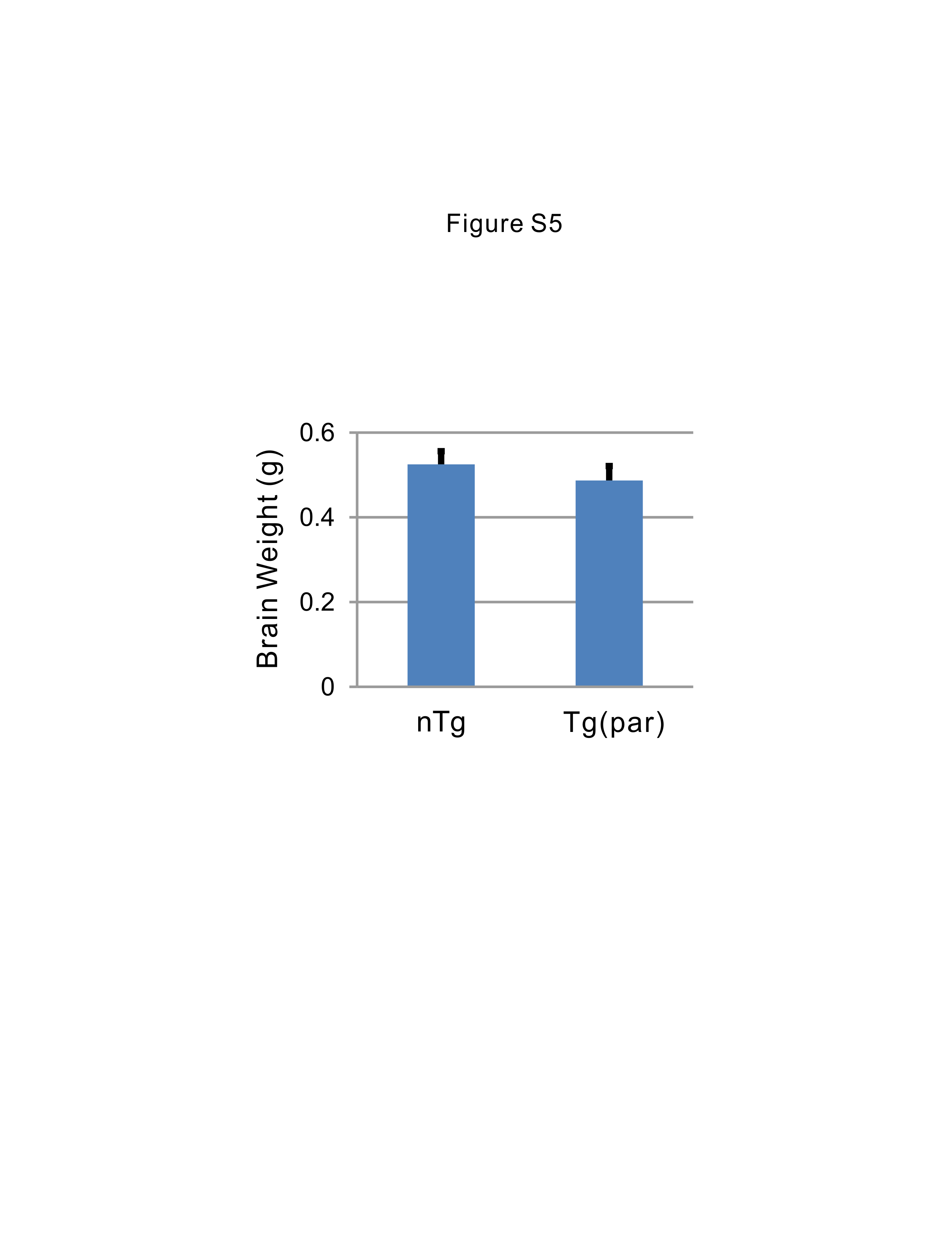

Supplement: S5 Fig — (JPG) [file pone.0255710.s005.jpg]

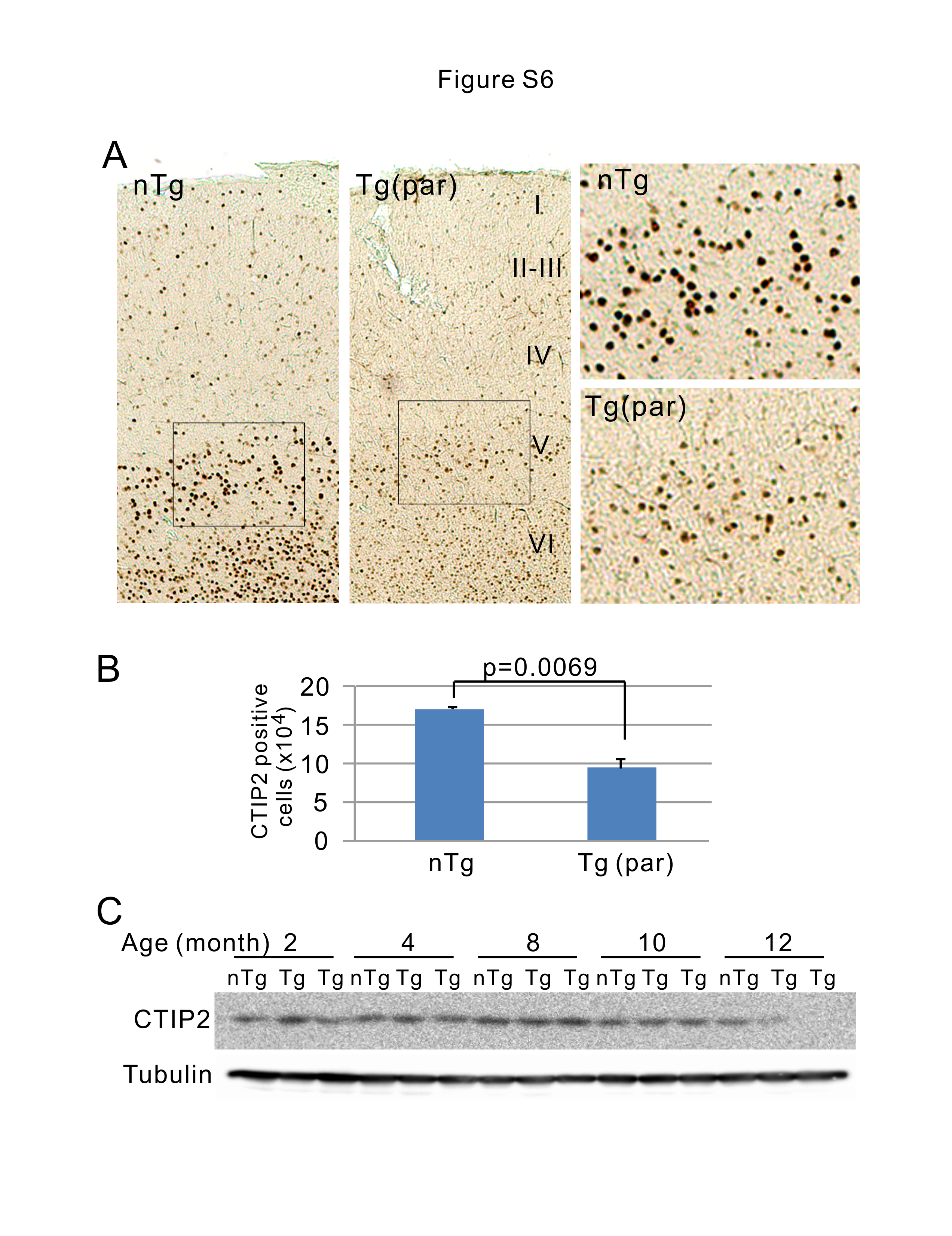

Supplement: S6 Fig — (A) CTIP2 staining of the motor cortex in nTg and paralyzed Tg mice. The boxed areas in layer V are enlarged in the two panels on the right. Notice a substantial reduction in the CTIP2 staining intensity in the Tg mice. (B) quantification of CTIP2-positive cells in layer V. (C) Protein blot of CTIP2 in the motor cortex from nTg and Tg mice at different ages. P-value was derived from Student’s t test. n = 4 for nTg and 3 for Tg mice. (JPG) [file pone.0255710.s006.jpg]

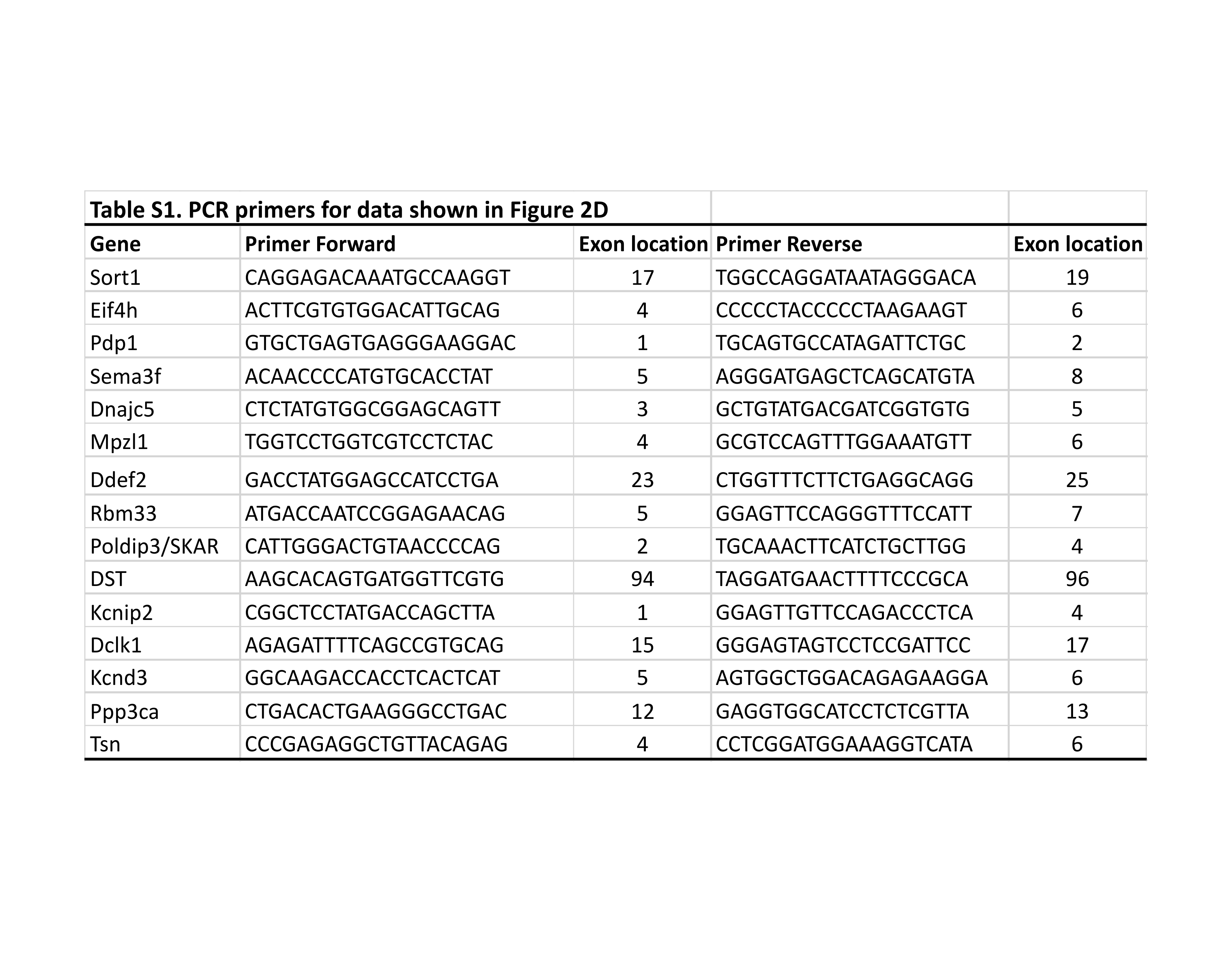

Supplement: S1 Table — (JPG) [file pone.0255710.s007.jpg]
