# Supplementary material for: Low-level overexpression of wild type TDP-43 causes late-onset, progressive neurodegeneration and paralysis in mice
Source: PLoS One. 2022 Feb 3;17(2):e0255710. doi: 10.1371/journal.pone.0255710 (PMC8812852; doi:10.1371/journal.pone.0255710)

Fig. 2A FB

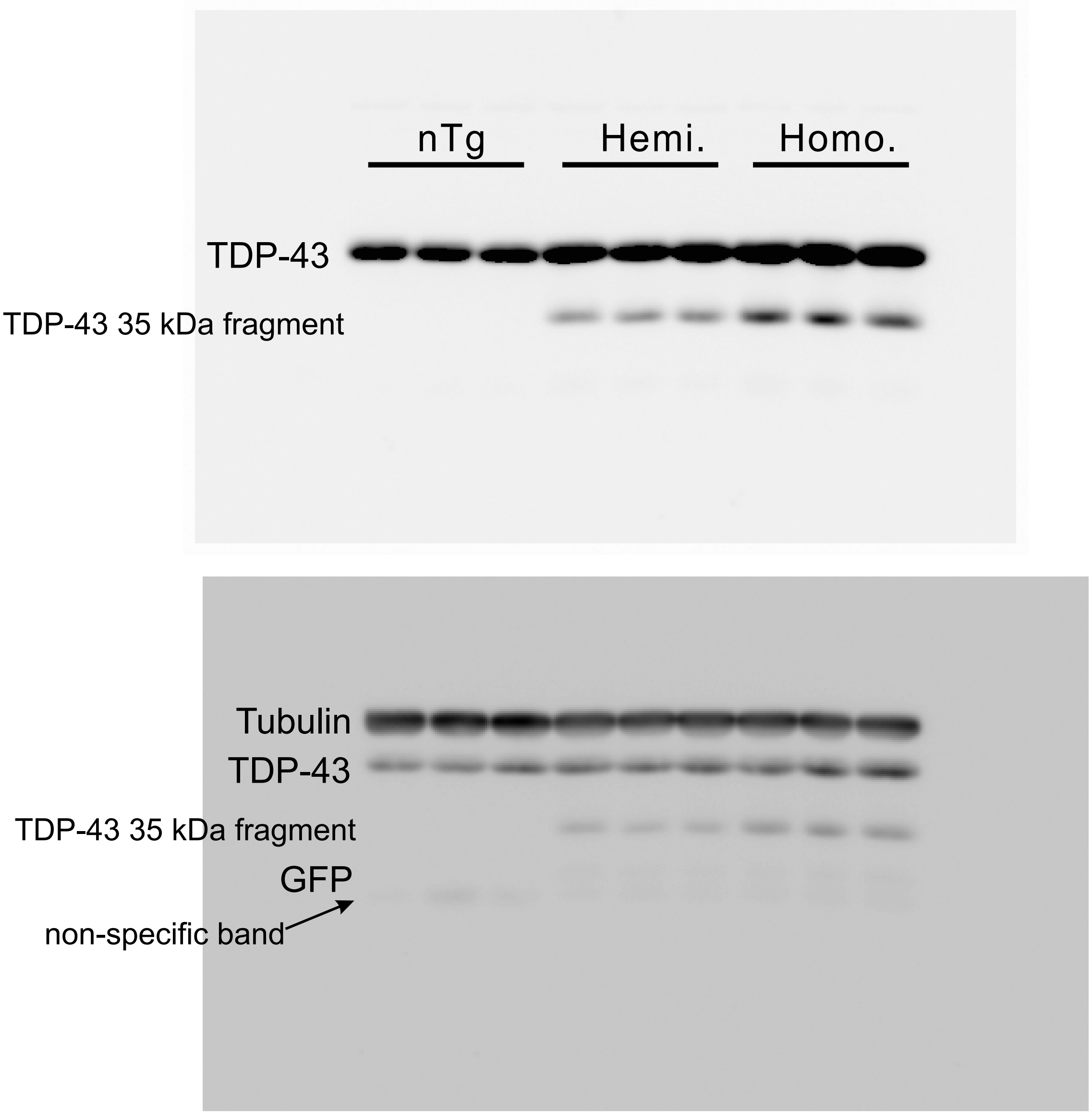

nTg
Hemi.
Homo.
TDP-43
TDP-43 35 kDa fragment
Tubulin
TDP-43
TDP-43 35 kDa fragment
GFP
non-specific band

Fig. 2A CB

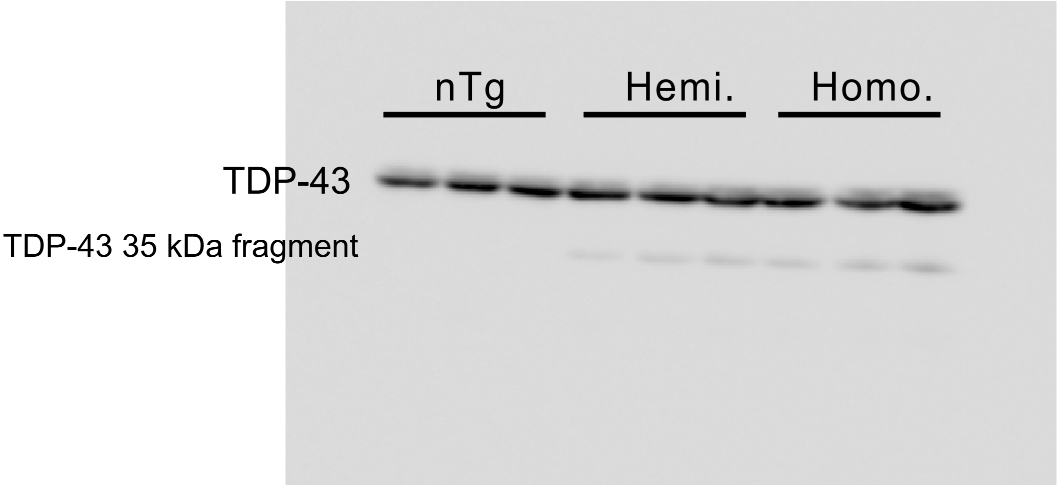
nTg
Hemi.
Homo.
TDP-43
TDP-43 35 kDa fragment

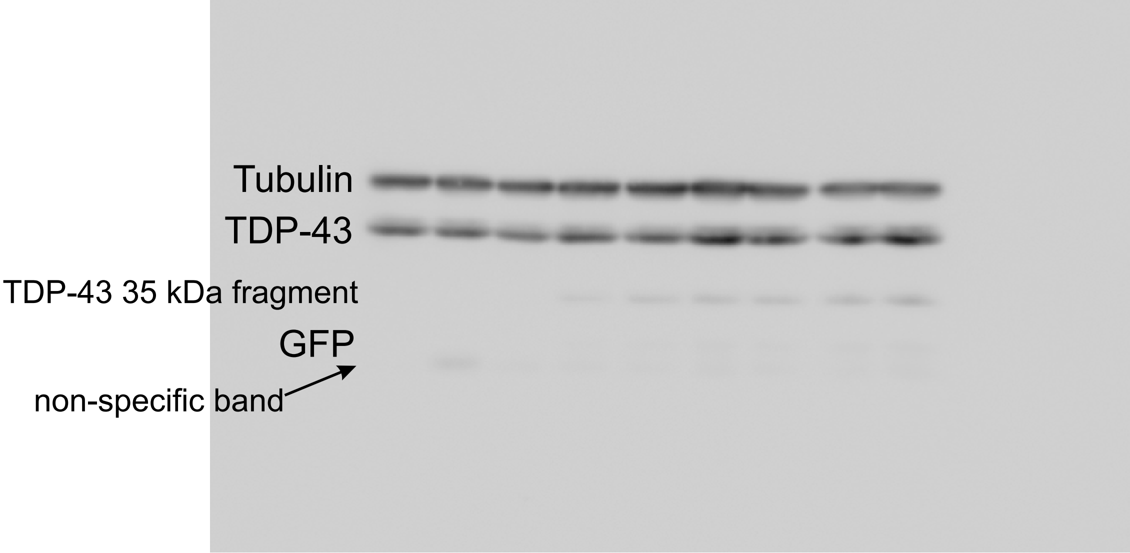
Tubulin
TDP-43
TDP-43 35 kDa fragment
GFP
non-specific band

Fig. 2A BS

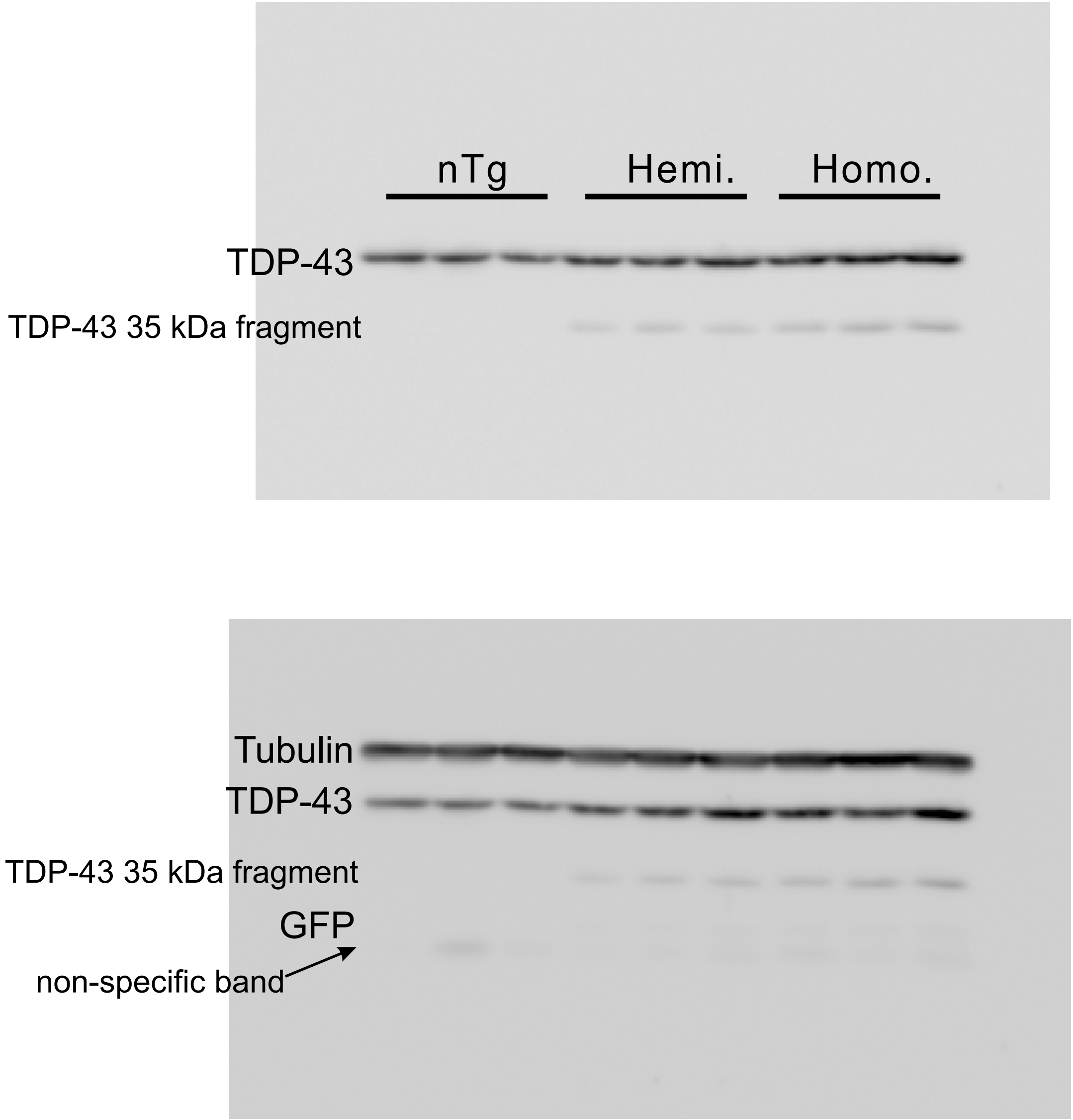

nTg
Hemi.
Homo.
TDP-43
TDP-43 35 kDa fragment
Tubulin
TDP-43
TDP-43 35 kDa fragment
GFP
non-specific band

Fig. 2A CSC

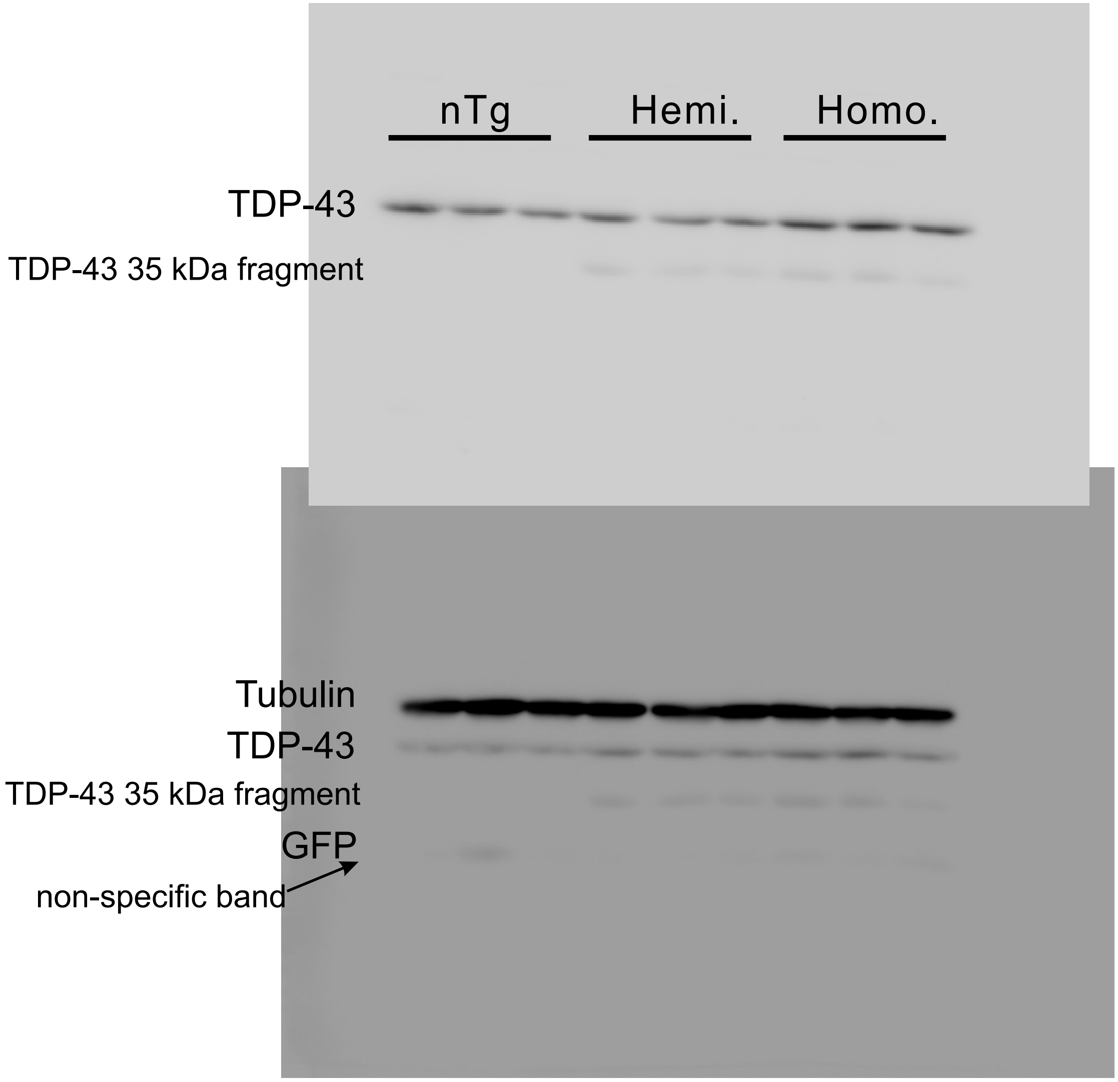

nTg
Hemi.
Homo.
TDP-43
TDP-43 35 kDa fragment
Tubulin
TDP-43
TDP-43 35 kDa fragment
GFP
non-specific band

Fig. 2A LSC

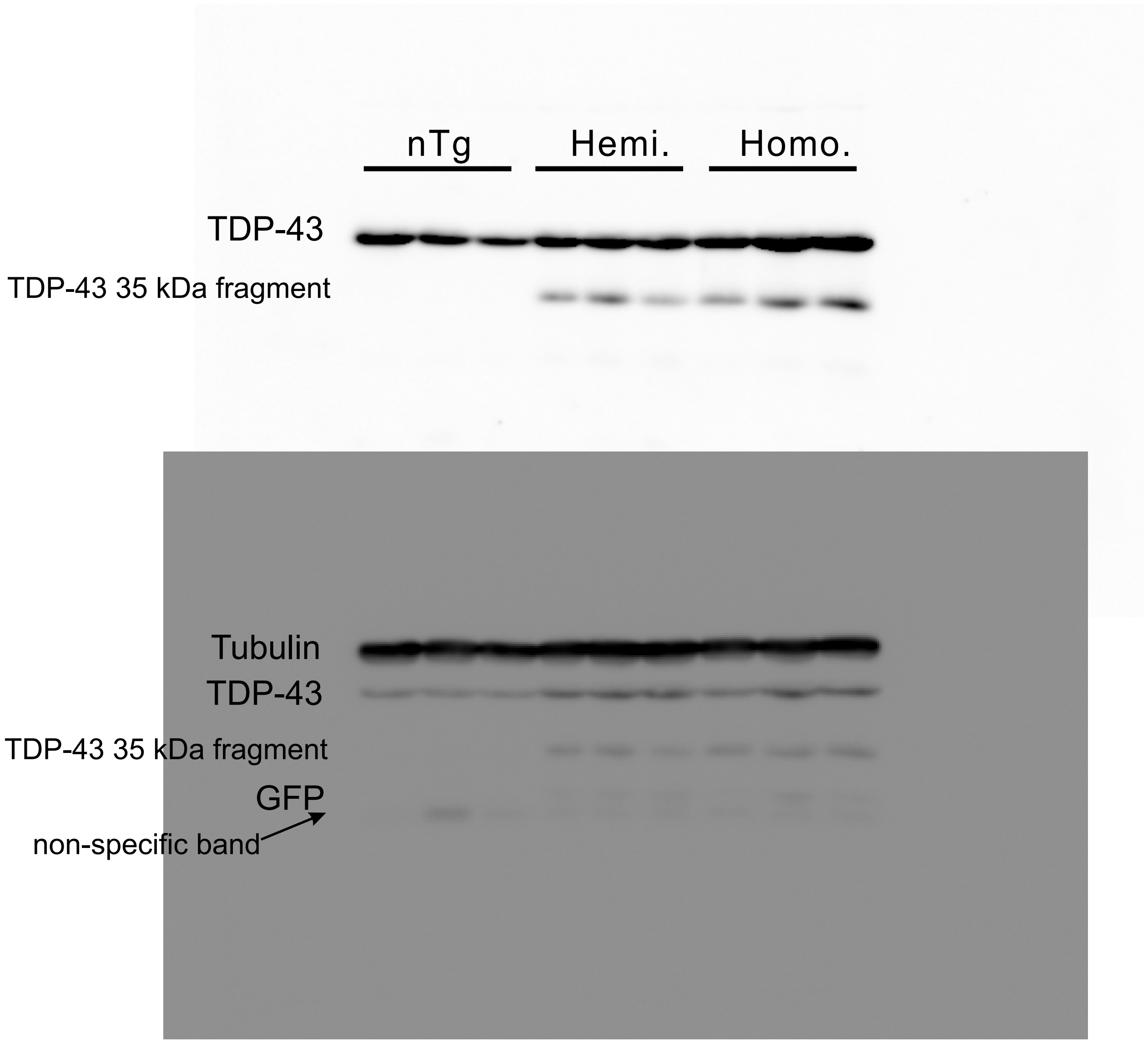

nTg
Hemi.
Homo.
TDP-43
TDP-43 35 kDa fragment
Tubulin
TDP-43
TDP-43 35 kDa fragment
GFP
non-specific band

Fig. 2F lumbar spinal cord

TDP-43

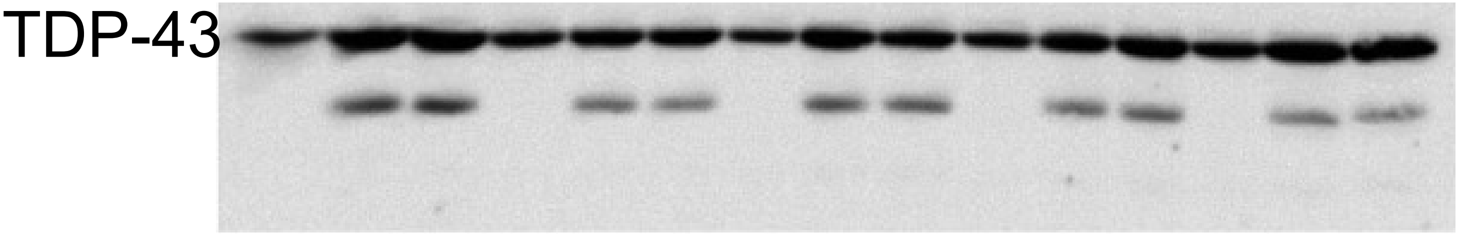

Note: The original blot could not be found. This is a cropped image that includes slightly larger area than the image shown in Fig. 2F.

Fig. 6B originals

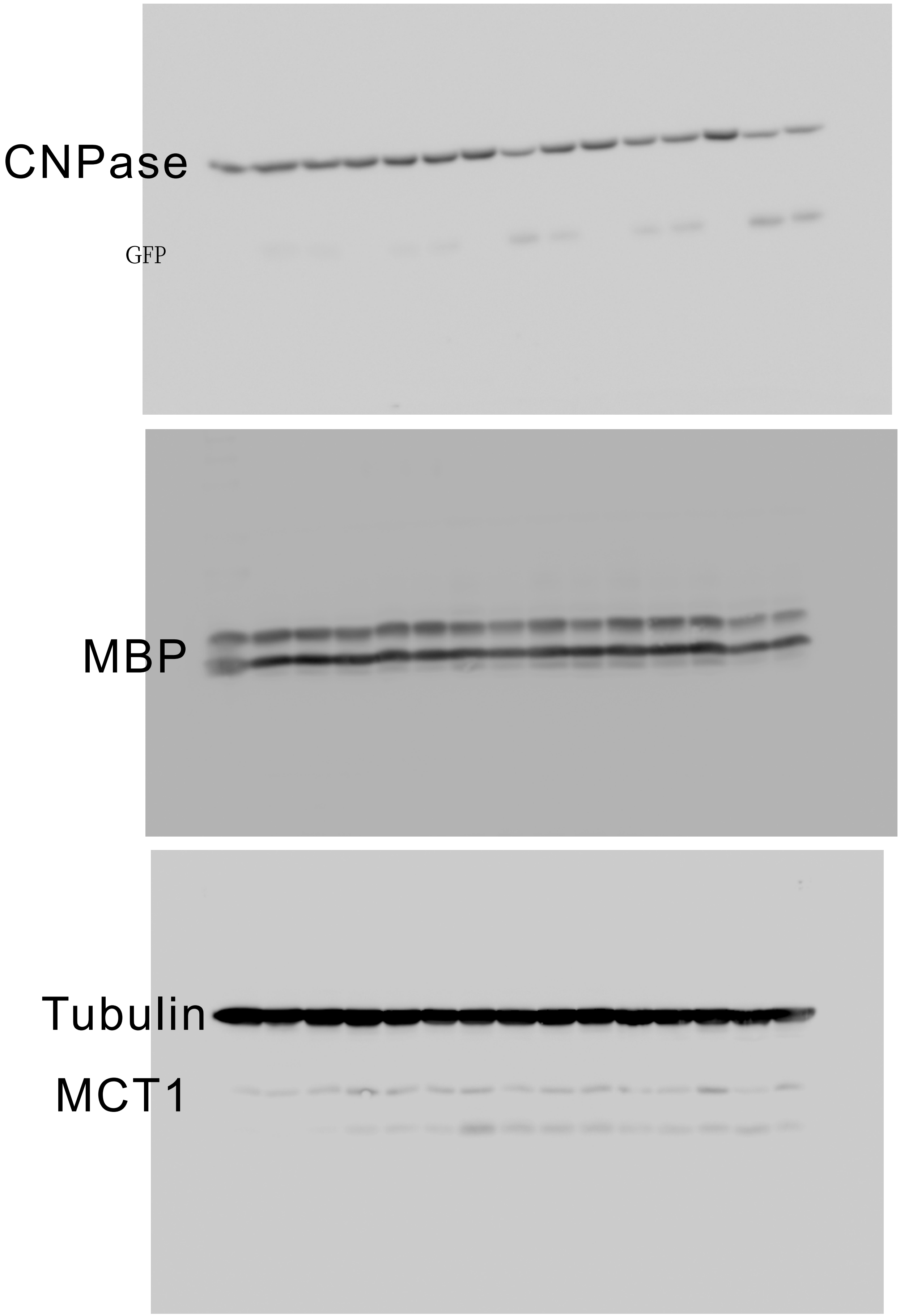

Fig 9B

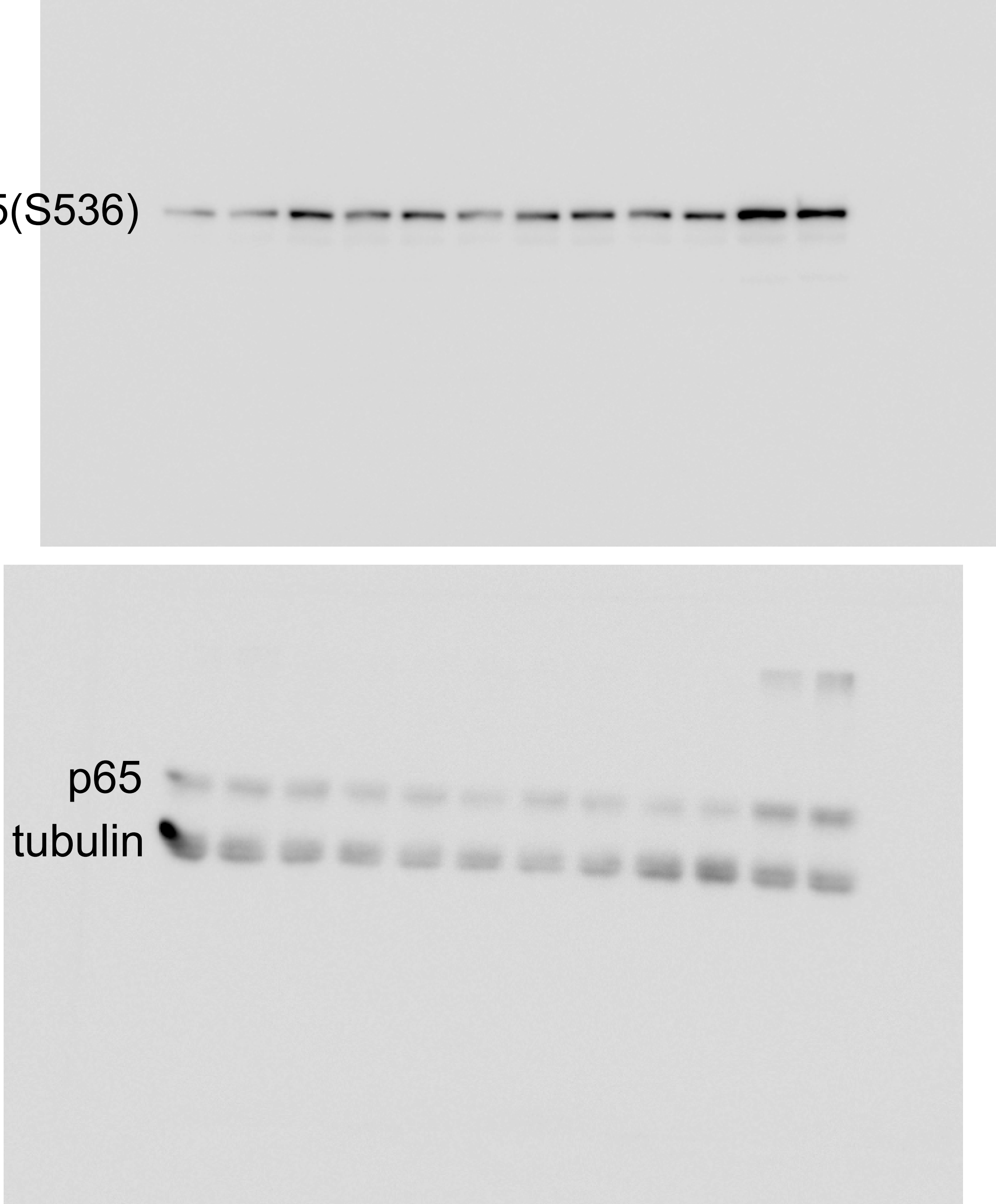

Fig. S1B

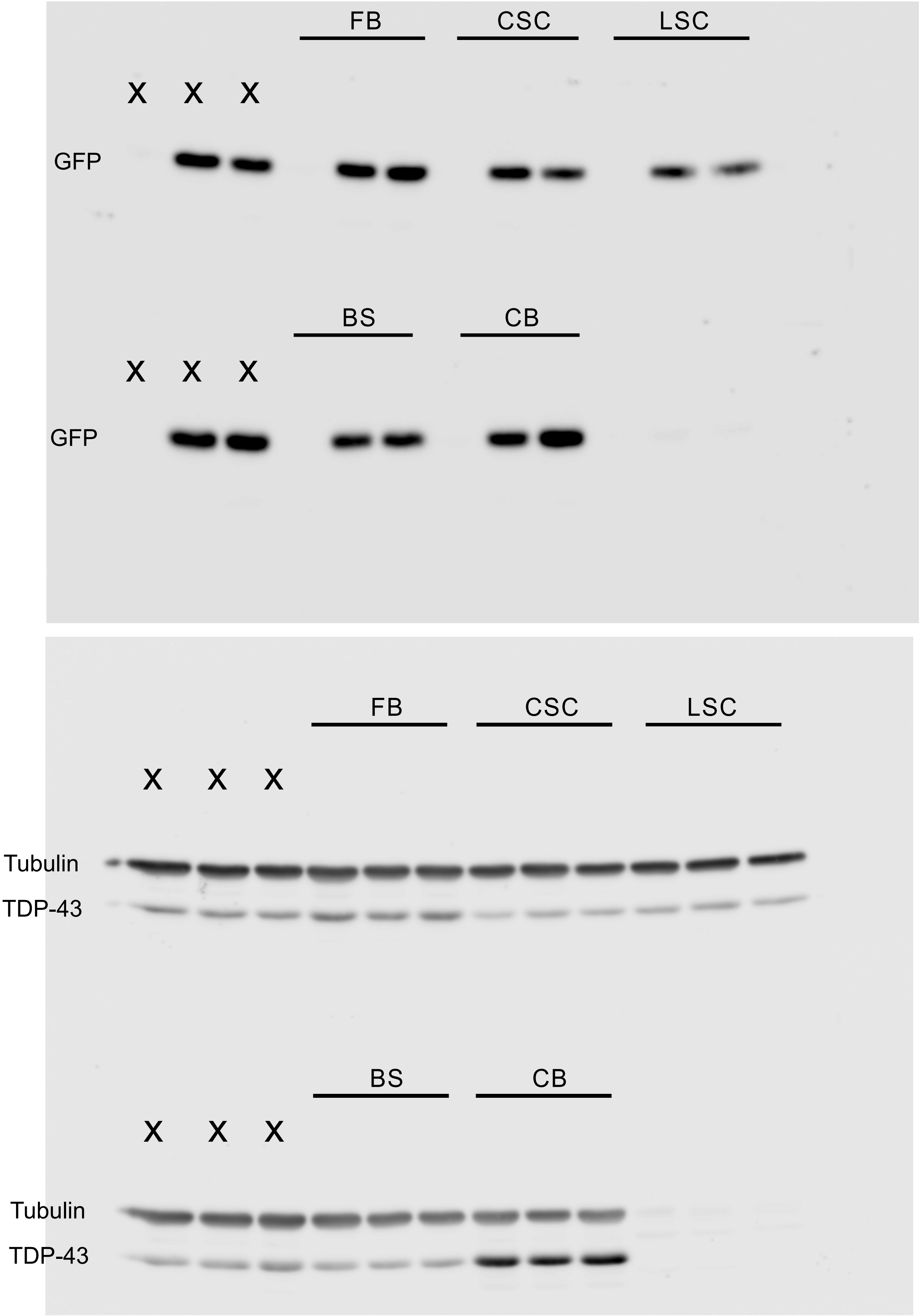
FB
CSC
LSC
X X X
GFP
BS
CB
X X X
GFP
FB
CSC
LSC
X X X
Tubulin
TDP-43
BS
CB
X X X
Tubulin
TDP-43

Fig. S1D

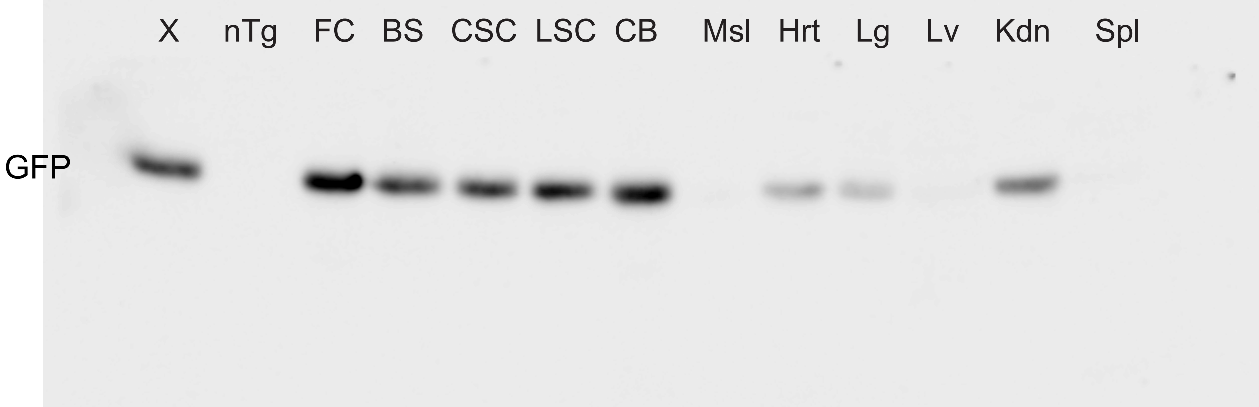

Fig. S1C

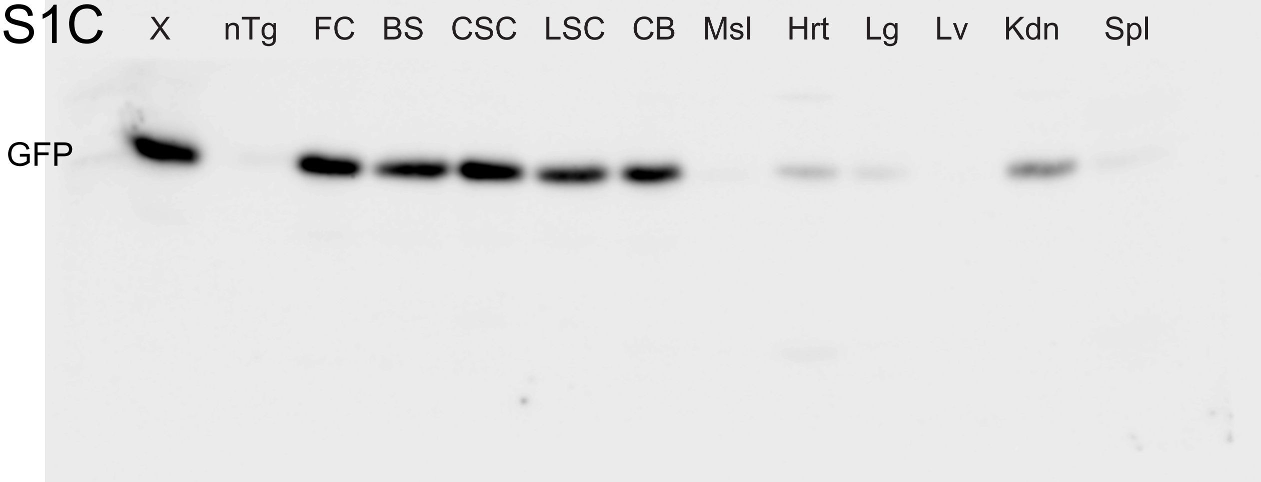

Fig. S1D

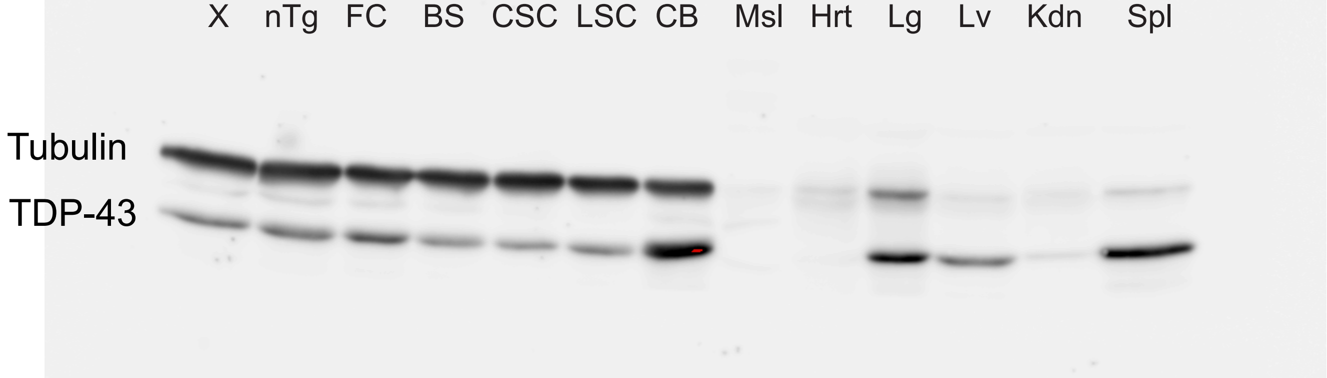

Fig. S1C

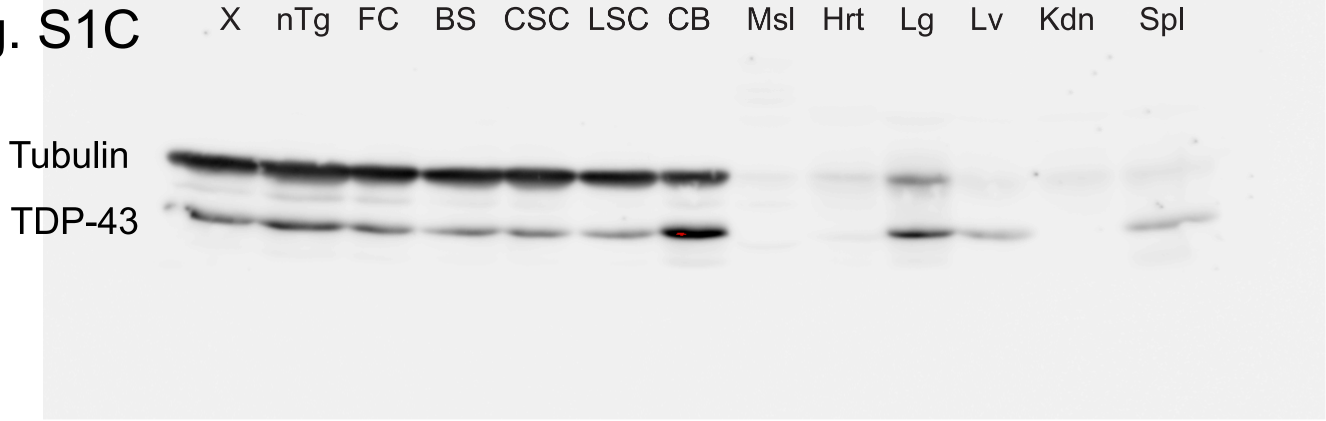

Fig. 2F lumbar spinal cord

TDP-43

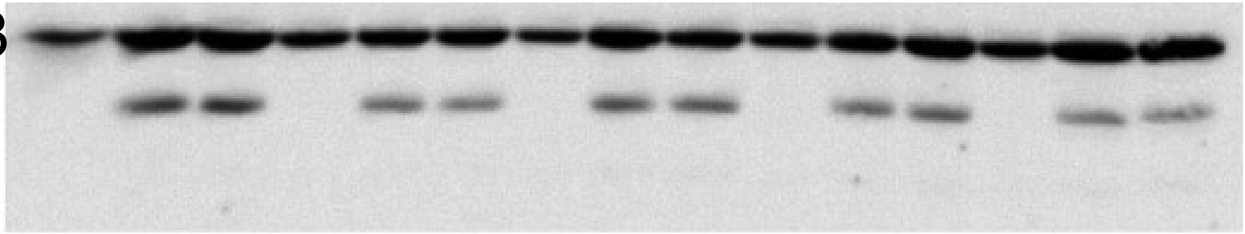

The original blot could not be found. This is a cropped image that contain a larger area of the blot than the one shown in Fig. 2F panel.

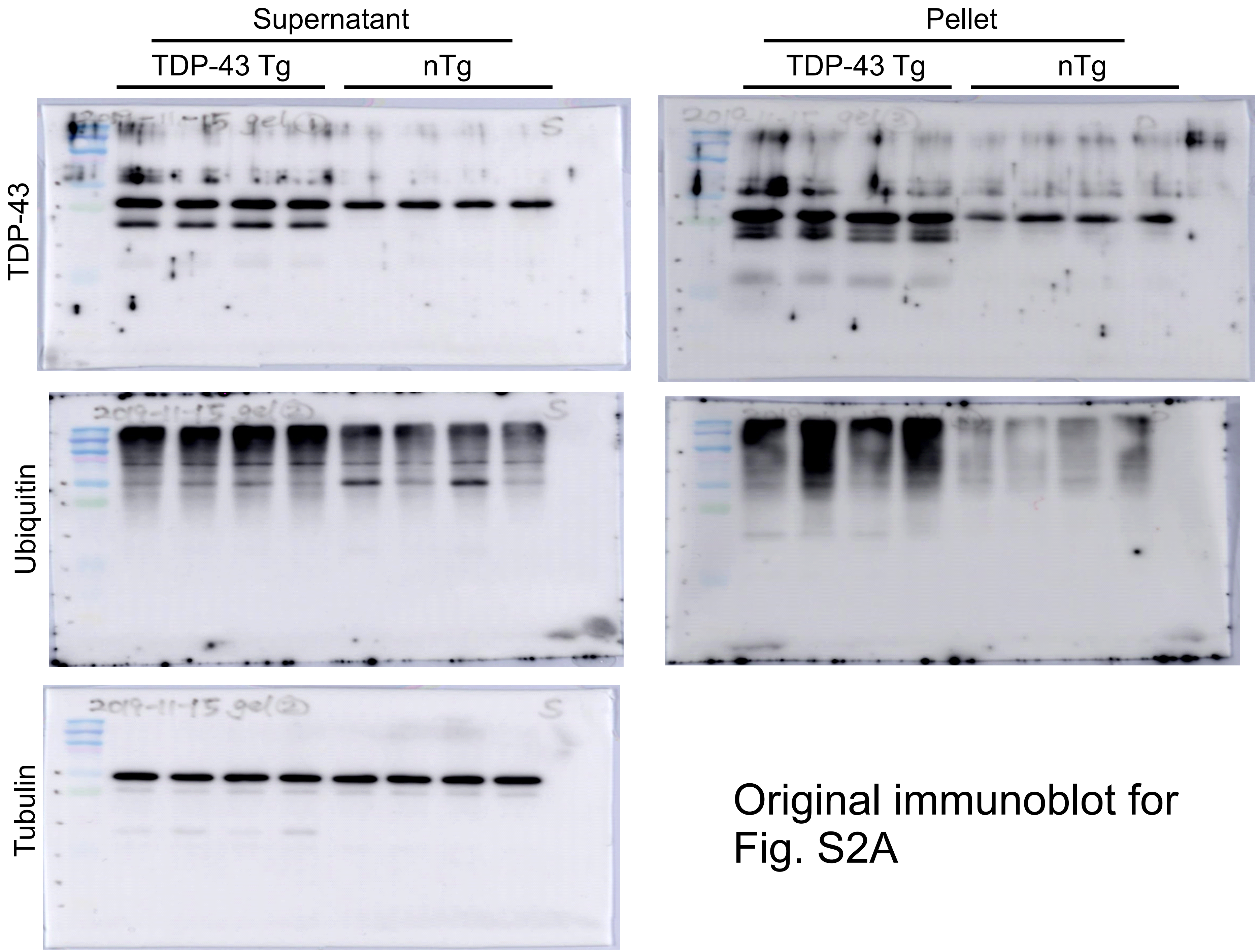

Original immunoblot for Fig. S2A

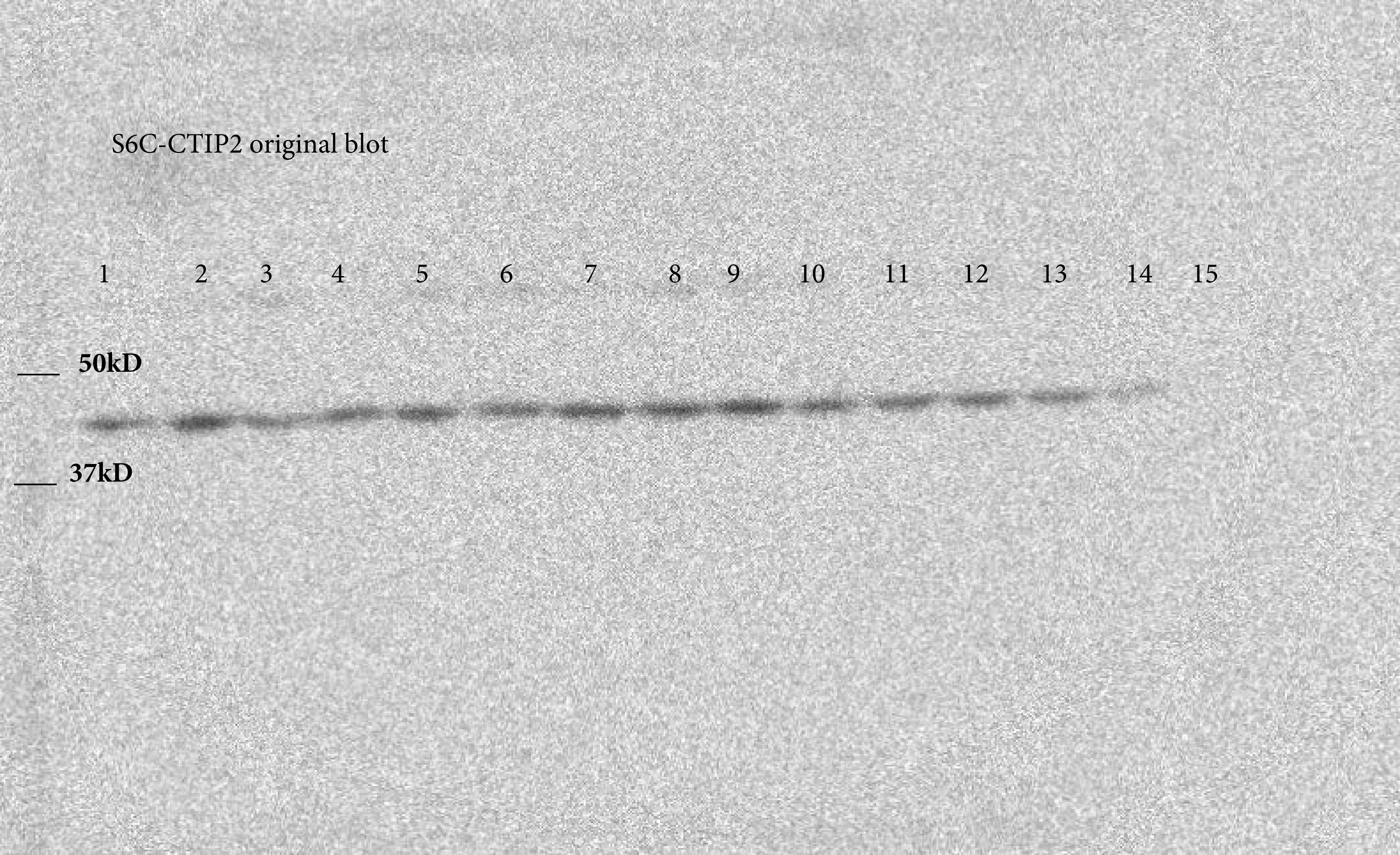
S6C-CTIP2 original blot
1 2 3 4 5 6 7 8 9 10 11 12 13 14 15
50kD
37kD

S6C- Tubulin original blot

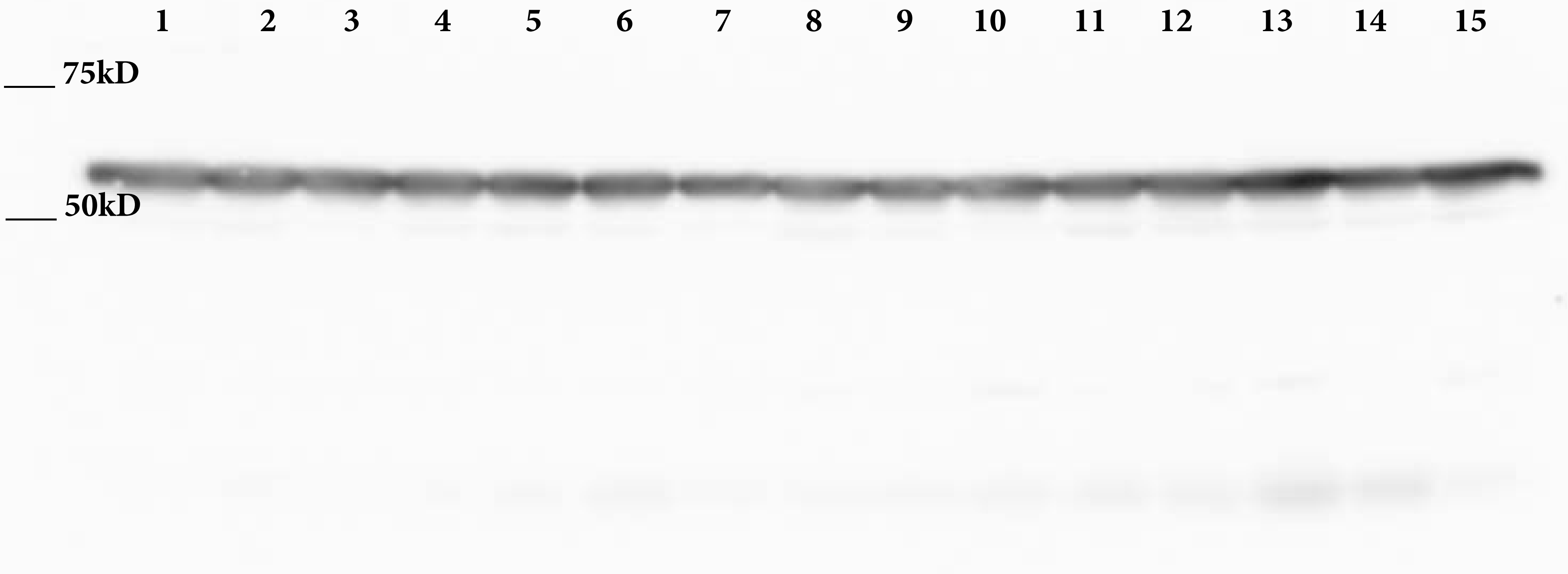

Supplement: S1 Raw images — (PDF) [file pone.0255710.s009.pdf]
